# From institutional trust to AI adoption: a trust transfer and risk perception model of AI-enabled public service acceptance in China's digital government context

**DOI:** 10.3389/fpsyg.2026.1860895

**Published:** 2026-07-28

**Authors:** Huihui Wang, Shixin Zhu

**Affiliations:** 1 School of Law and Public Administration, Hunan University of Science and Technology, Xiangtan, China; 2 Shandong College of Electronic Technology, Jinan, China

**Keywords:** AI service trust, digital government, institutional trust, LLM simulation, risk perception

## Abstract

**Introduction:**

Artificial intelligence (AI) is increasingly used in public agencies to route inquiries, screen eligibility, support caseworkers, and automate routine service encounters. Citizen acceptance of these services depends on their links to public authority, accountability, and visible opportunities for human recourse. This study examines a trust-based mechanism connecting institutional trust, risk perception, AI service trust, and behavioral intention in China's digital government context.

**Methods:**

The study combined an LLM-driven agent simulation involving 936 agents across three independent seeds, a 3 × 3 factorial scenario experiment involving 900 simulated agents, and a human-validation pilot using the same questionnaire and scenario structure. The pilot generated 189 submitted records, of which 182 were retained after attention checking.

**Results:**

In the synthetic calibration, institutional trust is positively associated with AI service trust (IT → AST β = 0.607) and negatively associated with risk perception (IT → RP β = −0.271); risk perception is negatively associated with AI service trust (RP → AST β = −0.459); and AI service trust is positively associated with behavioral intention (AST → BI β = 0.424). The same directional pattern appears in the human-validation pilot (IT → AST β = 0.357; IT → RP β = −0.240; RP → AST β = −0.513; AST → BI β = 0.650). Scenario means also align with the simulation pattern (Pearson *r* = 0.803 for AST and *r* = 0.875 for BI across the nine cells), with the lowest pilot AST (3.667) and BI (3.413) in the fully automated high-risk condition.

**Discussion:**

The findings connect confidence in government institutions with service-specific trust and indicate that perceived risk constrains acceptance of AI-enabled public services. In high-stakes automated settings, visible arrangements for human review may be necessary for AI service trust to translate into intended use. Public-sector AI acceptance is therefore shaped jointly by institutional credibility, perceived risk, and service encounter design.

## 1 Introduction

Public agencies increasingly use AI to organize citizen-facing service delivery, from routing inquiries and prioritizing inspection tasks to screening eligibility, assisting caseworkers, and powering government chatbots. These applications shift automated systems from administrative support into encounters where citizens evaluate public authority through a digital interface. Acceptance of public-service AI varies with the task being automated and the consequences attached to the decision (Gesk and Leyer, 2022; Horvath et al., 2023). In China's platformized digital government environment, where online service portals and integrated apps have become routine channels of administration, citizens are likely to judge AI-enabled services by their understandability, fairness, accountability, and fit with the stakes of the service (CNNIC, 2023). Transparency, ethical safeguards, and perceived benefits shape this confidence in government AI, especially when automated services move closer to consequential administrative decisions (Bian et al., 2025).

Once digital services begin to structure administrative judgment, the conventional adoption logic of e-government becomes less complete. Research on e-government established that trust, service quality, convenience, and user capability affect citizens' willingness to use online public services (Warkentin et al., 2002; Parent et al., 2005). Later studies added that digital literacy and citizen capability condition the movement from trust to use (Olesen et al., 2022; AbdulKareem and Oladimeji, 2024). AI-enabled public services bring another layer of uncertainty into this relationship. Citizens must consider how outputs are produced, how errors can be corrected, and where responsibility sits when automated systems influence a public service outcome.

Research on public-sector AI has moved this discussion beyond performance and convenience by emphasizing task risk, accountability, ethical deployment, and the continuing role of human judgment. Support for automation depends on whether a task is suitable for algorithmic processing and whether discretion remains visible when consequences become sensitive (Gesk and Leyer, 2022; Horvath et al., 2023). Ethical governance and institutional trustworthiness matter because public-sector AI operates under state authority (Kinder et al., 2023; Li, 2024). Studies of government chatbots sharpen the same point at the service interface: identity cues, escalation options, exception handling, and similarity to civil servants affect how citizens read an automated encounter (Larsen and Følstad, 2024; Li and Wang, 2024). A human official, a branded government chatbot, and a fully automated decision system communicate different expectations about empathy, recourse, and accountability (Wang et al., 2024; Senadheera et al., 2024). These differences make acceptance a question of institutional embedding as well as technical performance.

At the point where citizens evaluate an unfamiliar AI service delivered under state authority, institutional trust connects service-level judgments to the broader citizen–state relationship. Citizens often lack the technical knowledge needed to assess algorithmic quality directly, but they routinely hold beliefs about whether public institutions are competent, fair, and benevolent. Those beliefs can guide evaluations of unfamiliar technologies deployed by the state. Trust transfer theory explains how confidence in a familiar source can extend to a related target when the relationship between the two is salient (Stewart, 2003; McKnight et al., 2002). Evidence from digital health services shows a comparable pattern, where provider or platform trust supports acceptance of a novel service embedded in that setting (Meng et al., 2019). In government AI services, trust in public institutions can create an initial presumption of oversight, correction, and public responsibility.

Separate streams of research have clarified that e-government adoption depends on a trust foundation (Parent et al., 2005; Olesen et al., 2022) and that public-sector AI acceptance is conditioned by transparency, safeguards, and task context (Horvath et al., 2023; Bian et al., 2025). The unresolved issue is more precise: how general confidence in government enters citizens' evaluation of a particular AI-enabled public service. Institutional trust, perceived risk, AI service trust, and behavioral intention are often studied as adjacent factors, yet the acceptance problem is a chain of judgments. Institutional confidence may support service trust, perceived risk may weaken that trust, and the presence or absence of human review may alter whether trust becomes willingness to use the service.

To address this mechanism, the article examines how institutional trust, risk perception, AI service trust, and behavioral intention are connected under different combinations of service format and risk level. The mechanism is traced through LLM-based simulation under comparable scenario conditions and through human-validation evidence assessing whether the same ordering appears in observed responses. Persona-conditioned language models make the theoretical assumptions explicit and keep the scenario manipulations comparable across conditions, while comparison with external human data strengthens interpretation of synthetic social simulation (Argyle et al., 2023; Bisbee et al., 2024). A human-validation pilot with the same 3 × 3 scenario structure examines whether the directional relationships found in the simulation also appear in participant responses (Aher et al., 2023; Bail, 2024).

## 2 Literature review and theoretical development

### 2.1 Institutional trust and AI-enabled digital government

Before citizens evaluate a particular digital service, institutional trust gives them a prior judgment about whether public authorities are competent, procedurally fair, and oriented toward citizens' interests. In digital government, this judgment matters because citizens use public services through systems whose data practices, decision routines, and administrative safeguards are partly invisible from the user side. Early e-government research established trust as a condition for online public-service adoption (Warkentin et al., 2002; Parent et al., 2005). Later work shows that digital literacy, service experience, and citizen capability affect whether confidence in digital government becomes actual use (Olesen et al., 2022; AbdulKareem and Oladimeji, 2024). When AI is embedded in public service delivery, institutional trust supplies a credibility frame before citizens can observe the system's technical operation in detail.

When classification, recommendation, and decision support enter the citizen–state encounter, AI changes the meaning of digital-service acceptance. A usable interface can improve access, but it tells citizens little about whether an agency can govern an algorithm responsibly, correct a wrong output, or remain accountable for the consequences of automated processing. Public-sector AI research shows that citizens attend to task appropriateness, explainability, accountability, and institutional recourse when evaluating automated services (Gesk and Leyer, 2022; Horvath et al., 2023). Trustworthiness cues attached to the deploying institution also shape public confidence in government AI (Li, 2024; Bian et al., 2025). Service credibility, governability, and responsibility are judged through both the technology and the public authority that deploys it.

In China's platformized digital government environment, the connection between institutional credibility and service evaluation is especially visible. Citizens increasingly encounter the state through integrated digital interfaces, and AI is discussed in relation to screening, routing, recommendation, and adjudication-related support. As automation moves closer to consequential functions, acceptance depends on more than whether the service works quickly. Citizens are also likely to ask whether public responsibility remains identifiable and whether the technology appears governable within the administrative system.

### 2.2 Trust transfer as the mechanism linking government trust to AI service trust

To explain how general confidence in government reaches a specific AI-enabled service, trust transfer provides the mechanism connecting the institutional source to the service target. Zucker's account treats trust as produced through stable organizational arrangements, and Mayer et al. (1995)'s integrative model specifies the evaluative content of trust in terms of perceived ability, benevolence, and integrity (Zucker, 1986). These dimensions are initially attached to government as the institution responsible for service delivery. When an AI system is presented as part of that service infrastructure, institutional confidence can inform citizens' expectations about whether the service is competent, appropriately governed, and aligned with public purposes.

Within this source-target relation, trust transfer theory explains how confidence moves from a familiar institution to a related service object. Stewart emphasizes the source-target structure of the transfer, and McKnight et al. (2002) show that the relationship becomes more influential when the connection between the source and target is salient (Stewart, 2003). Evidence from digital and health-service settings shows a similar pattern: trust in a provider, platform, or institutional arrangement can support acceptance of a novel service embedded in that environment (Meng et al., 2019). In AI-enabled public services, the source is government and the target is the AI service operating under government authority. Because most citizens cannot directly assess algorithmic quality, institutional cues become part of how service judgments are formed.

For AI-enabled public services, the content of service trust extends beyond a general preference for technology use. It refers to a service-level judgment about whether an AI-enabled public service is competent, useful, stable, task-appropriate, and institutionally governable. Research on trust in automation supports the reliance component of this definition (Lee and See, 2004; Glikson and Woolley, 2020), and technology-trust research treats trust as an evaluation of system attributes distinct from a simple behavioral inclination (Lankton et al., 2015). Public-sector AI research adds the governance dimension, because citizens also consider whether the service remains accountable within public administration (Li, 2024). AI service trust remains a service-specific judgment, even when it draws on confidence in the institution behind the service.

Once citizens encounter the service interface, the strength of transfer depends on whether the institutional source remains visible. A government chatbot can make the institutional source visible through branding, language, and escalation pathways, whereas a fully automated decision system may make human oversight and responsibility less apparent. Studies of permit automation and government chatbots show that service format, discretion, and perceived accountability alter citizen evaluations of public-sector AI (Horvath et al., 2023; Wang et al., 2024; Senadheera et al., 2024). Trust transfer in this context depends on how clearly the AI service remains tied to public authority, oversight, and recourse.

### 2.3 Risk perception, human involvement, and boundary conditions of public-sector AI acceptance

As automated systems move into public-service tasks with administrative consequences, risk perception translates governance uncertainty into service-level evaluation. Following Featherman and Pavlou (2003), perceived risk is treated as a multidimensional expectation of possible loss . In AI-enabled public services, such losses can arise from user-side conditions such as digital literacy and the ability to challenge an output, administrative conditions such as data governance and responsibility allocation, and technology-side conditions such as opacity, model error, privacy exposure, or biased classification. Welfare, complaint handling, eligibility review, and enforcement-related services make these risks especially salient because procedural fairness and correction channels are part of the service itself.

When automation is applied to sensitive administrative tasks, public support depends on whether the task appears suitable for algorithmic processing and whether the system remains visibly supervised. Gesk and Leyer (2022) emphasize task boundedness and appropriateness, and Horvath et al. (2023) show that support declines as automation moves into sensitive permit decisions. Research on algorithmic government and facial-recognition policing reaches a similar conclusion by showing that institutional trustworthiness and perceived governance conditions shape public support (Sidhu et al., 2024; Li, 2024). Concerns about governance, fairness, and contestability are part of the service evaluation itself, not general reactions to technology.

Within AI-mediated service encounters, human involvement gives citizens a visible cue about responsiveness and responsibility. A human official, a government chatbot, and a fully automated decision system differ in the degree to which they signal empathy, recourse, discretion, and administrative accountability. Virtual-agent research explains why automated interfaces can create service value when they are useful and easy to access (Scutella et al., 2022). Government chatbot research places additional weight on identity cues, escalation pathways, exception handling, and visible human oversight (Larsen and Følstad, 2024; Li and Wang, 2024; Wang et al., 2024). Communicative warmth and perceived empathy also shape trust in AI-mediated public-service encounters (Zhou et al., 2025). The contrast among human officials, government chatbots, and fully automated decision systems captures different levels of visible discretion and recourse.

In higher-stakes settings, algorithm-aversion research clarifies why service risk and automation level should matter together. Dietvorst et al. (2015) show that confidence in algorithmic advice often declines once the possibility of error becomes salient, and later work extends this pattern to contexts involving judgment, morality, and sensitivity (Logg et al., 2019; Bigman and Gray, 2018). Public services bring these concerns into an institutional setting: a high-risk service delivered by a fully automated system offers fewer visible cues of human discretion and recourse than a human official or a chatbot with escalation possibilities.

### 2.4 Research model and hypotheses

**Figure 1:**
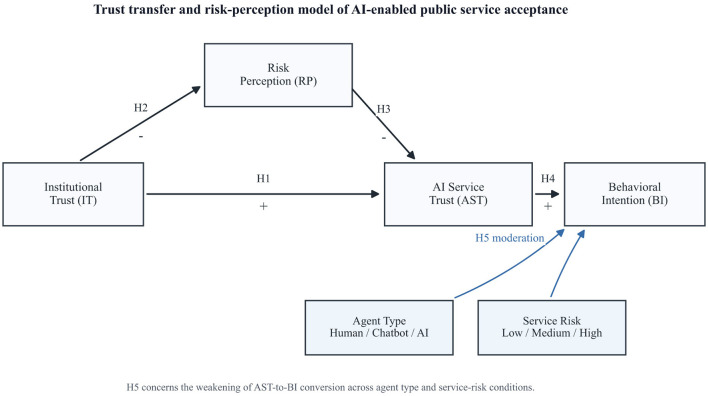
Conceptual path model of institutional trust, risk perception, AI service trust, and behavioral intention.

Building on these arguments, the proposed model links institutional trust to behavioral intention through two service-level judgments: perceived risk and AI service trust (Figure 1). Institutional trust is placed at the source of the chain because it supplies the initial credibility frame for a government AI service. Risk perception captures the governance-uncertainty route through which concerns about opacity, error, responsibility, and recourse reduce service confidence. AI service trust represents the proximate judgment that the service is competent, useful, and governable, and behavioral intention captures willingness to rely on the service.

When an AI system is presented as part of public service delivery, confidence in government should support an initial belief that the service is being deployed under acceptable administrative conditions. H1. Institutional trust is positively associated with AI service trust.

When institutional confidence signals oversight, procedural safeguards, and channels for correction, perceived risk should decrease in an automated service encounter. H2. Institutional trust is negatively associated with risk perception.

At the next step in the acceptance judgment, perceived risk connects governance concerns to service-level trust. Concerns about user capability, data governance, privacy, model error, procedural unfairness, or weak recourse should lower confidence that the AI service is suitable for public administration. H3. Risk perception is negatively associated with AI service trust.

Service-level trust should then increase willingness to use the AI-enabled service when citizens regard it as a usable and accountable channel for completing a public-service task. Technology-adoption research treats confidence in a service as a proximate condition for behavioral willingness (Davis, 1989; Venkatesh et al., 2003). H4. AI service trust is positively associated with behavioral intention.

Because service risk and automation level change the visibility of discretion and recourse, the scenario condition specifies where this final link is expected to weaken. A fully automated high-risk service gives citizens fewer visible cues of human discretion, empathy, and recourse than a chatbot or human official. H5. Under fully automated and high-risk conditions, the positive relationship between AI service trust and behavioral intention is weaker than under chatbot or human-official conditions.

In substantive terms, the solid paths in the model represent the main trust chain from institutional confidence to service-level trust and intended use. The scenario condition attaches the chain to public-service context: the conversion of AI service trust into behavioral intention is expected to be weakest when automation is complete and the service carries higher risk.

## 3 Research design and methods

### 3.1 Research design and analytical scope

To examine the proposed mechanism across controlled scenarios and observed responses, the design separates mechanism tracing, scenario comparison, and external alignment. A main synthetic simulation with *n* = 936 LLM-generated agents across three independent random seeds formalizes the proposed trust chain and inspects whether it produces the expected calibration pattern under controlled assumptions. A 3 × 3 synthetic scenario experiment with *n* = 900 agents varies service agent type and service risk level to examine where the trust-to-use-intention link weakens. A supplementary human-validation pilot retains the same scenario structure and questionnaire to compare the synthetic ordering with observed responses (189 submitted records; 182 valid responses after attention checking).

The synthetic component formalizes the model assumptions under controlled conditions and creates scenario contrasts that remain comparable across service format and risk level. The human-validation pilot compares those patterns with questionnaire responses from public-administration graduate students and part-time MPA students experienced in digital public services.

China is an appropriate analytical context because public services are highly platformized and AI applications are increasingly discussed in relation to administrative coordination, service routing, eligibility assessment, and decision support. Users often interpret AI services through the authority and accountability of the institution that deploys them (CNNIC, 2023; Bian et al., 2025). The setting also brings the digital-divide problem into view: older, rural, less educated, and lower-income groups may experience AI-enabled services differently because access, digital literacy, and confidence in self-service systems are unevenly distributed.

### 3.2 Synthetic persona construction and calibration boundary

Synthetic personas varied age, gender, education, occupation, residence or city tier, and prior experience with e-government services. Seed values were calibrated with reference to the Seventh National Population Census for age, residence, and education distributions, and to the CNNIC report for internet use and digital public-service exposure (National Bureau of Statistics of China, 2021; CNNIC, 2023). The persona frame gives greater weight to urban and digitally experienced public-service users. Table 1 summarizes the calibration sources and interpretive scope for the persona dimensions.

**Table 1:** Persona calibration sources and interpretive scope.

Persona dimension	Calibration source	Interpretive scope
Age and residence	Seventh National Population Census and CNNIC internet-user profile	Adults aged 18–65 were stratified by age band, with higher weights for urban and county/town digital-service users.
Education	Seventh National Population Census and CNNIC digital-user indicators	Personas with a bachelor's degree or higher account for approximately 57% of the synthetic frame, representing a digitally active segment of public-service users.
Occupation and public-sector contact	Public-service-use relevance and scenario fit	Profiles include public-sector employees, private-sector employees, self-employed workers, students, retirees, and users with varied e-government experience.
Digital experience	CNNIC internet and digital-service context	Digital literacy and prior e-government use were varied across personas who can complete online public-service interactions.

Age and residence anchor the persona frame in census and internet-user distributions; education, occupation, and digital experience specify the service-user profile used in the synthetic simulation. The resulting frame is centered on digitally active users who are likely to encounter online public-service automation.

Anthropic's Claude Opus 4.6 model (claude-opus-4-6) served as the simulation engine for persona-conditioned scenario responses. Prompt templates anchored each response in assigned persona attributes, introduced both favorable and unfavorable reactions to the service, and withheld hypotheses and expected coefficient signs from the response prompt. Independent seeds, randomized scenario assignment, and temperature sensitivity checks reduced dependence on a single generation run and limited obvious expectation alignment.

### 3.3 Measurement

Because the theory depends on the movement from institutional confidence to service-level acceptance, the measurement model distinguishes institutional, risk, service-trust, and behavioral-intention judgments before the scenario manipulation is introduced. Institutional trust (IT) refers to confidence in the public institution providing the service, including competence, benevolence, fairness, and accountability. Risk perception (RP) captures concerns about user capability, opacity, privacy, procedural fairness, misuse, error consequences, and recourse. AI service trust (AST) refers to a service-level judgment that the AI-enabled service can understand the user's request, provide useful guidance, perform consistently, fit the administrative task, and remain institutionally governable. Behavioral intention (BI) captures willingness to use or rely on the service in a comparable real-world context.

Measurement items were adapted from adjacent research areas because no established scale fully matches the present combination of institutional trust, public-service AI, and scenario-based automation. Institutional trust items draw on public-sector trust and e-government adoption research (Parent et al., 2005; Mensah and Mi, 2017; Hartanti et al., 2021). Risk-perception items follow perceived-risk research and extend it to fairness, opacity, and accountability concerns specific to public-sector AI (Featherman and Pavlou, 2003; Li, 2024). AI service trust items combine the reliance logic of trust-in-automation research with public-service concerns about task fit, usefulness, institutional anchoring, and empathy in automated encounters (Lee and See, 2004; Glikson and Woolley, 2020; Zhou et al., 2025). Behavioral intention items follow the measurement logic of information-systems adoption research (Davis, 1989; Venkatesh et al., 2003). All core items use a 7-point Likert scale.

### 3.4 Synthetic simulation and scenario procedure

Within the synthetic simulation, each persona is represented through latent construct scores derived from persona-conditioned seed values and directional calibration rules corresponding to H1–H4. A factor-analytic measurement routine maps those latent scores into bounded 7-point item responses. Structural coefficients and item-loading targets are handled as simulation parameters. Synthetic coefficients function as calibration-consistent associations, and AVE, CR, and HTMT values function as measurement calibration diagnostics. Common reliability and discriminant-validity thresholds provide reference points for the synthetic measurement profile (Hair et al., 2019; Henseler et al., 2015).

To examine boundary conditions, the 3 × 3 scenario experiment varies service agent type and service risk level so that the same trust chain can be observed under different degrees of human involvement and task consequence. Agent type has three conditions: Human Official, Government Chatbot, and AI Decision System. The Human Official condition represents support from a public-sector employee. The Government Chatbot condition represents an AI assistant with clear government institutional branding and possible escalation. The AI Decision System condition represents a fully automated decision process without salient human oversight. Service risk also has three levels: low risk for routine information inquiry, medium risk for standard administrative transactions, and high risk for consequential eligibility or enforcement decisions. Each synthetic cell contains 100 agents.

Composite-score and standardized-association summaries keep the path diagnostics comparable across the three random seeds. Scenario-level AST and BI means, together with AST-to-BI associations, identify where the trust-to-use link weakens across the nine conditions.

Stability assessment relied on a pilot batch of 100 synthetic agents, three independent random seeds (42, 123, and 456; 312 agents per seed), and a temperature sensitivity check at *T* = 0.3 and *T* = 1.0 with 200 agents per condition.

### 3.5 Human-validation pilot

The external pilot retained the same 3 × 3 scenario structure and the same IT, RP, AST, and BI items, allowing the synthetic scenario ordering to be compared with responses from an educated public-administration sample. The sample consisted of graduate students in public administration, administrative management, public policy, emergency management, and related MPA programs. Random assignment placed each participant in one scenario cell. Background measures included age, gender, student status, residence origin, public-sector experience, prior e-government use, AI familiarity, and self-rated digital literacy. Seven records failed the attention check, leaving 182 valid responses for analysis. Valid cell sizes ranged from 18 to 21.

The validation logic rests on three comparisons: whether the adapted items show internal consistency in human responses, whether the H1–H4 paths retain their expected directions, and whether the nine-cell human scenario ordering aligns with the synthetic scenario pattern.

## 4 Results

### 4.1 Synthetic calibration diagnostics

**Figure 2:**
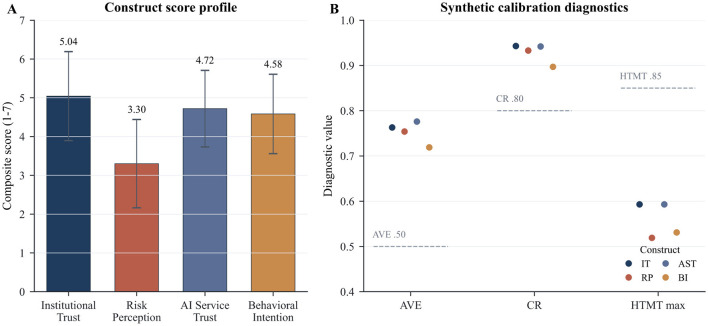
Synthetic measurement profile and calibration diagnostics. Panel **(A)** presents construct means with standard-deviation error bars on the 7-point response scale. Panel **(B)** presents AVE, CR, and maximum HTMT values as synthetic calibration diagnostics. Dashed reference guides indicate common thresholds for AVE, CR, and HTMT.

The synthetic calibration produces a differentiated response profile before the structural paths are examined. Institutional trust occupies the highest position in the generated responses, providing the upstream condition for trust transfer (Figure 2). Risk perception remains in the middle of the response range, which preserves a visible risk component within the simulated public-service judgment. AI service trust and behavioral intention also sit in the intermediate range.

The AVE, CR, and HTMT values reinforce this construct separation. The synthetic measurement profile shows sufficient internal consistency and discriminant structure for the subsequent path analysis under the specified loading and error-variance rules.

### 4.2 Synthetic path and scenario patterns

**Figure 3:**
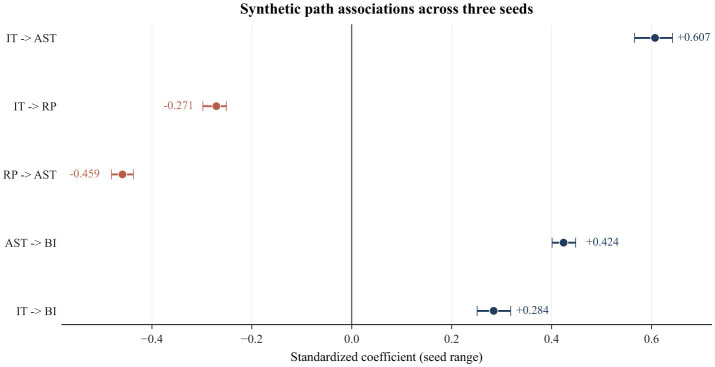
Synthetic path associations across three independent random seeds. Points indicate mean standardized coefficients and horizontal bars indicate seed ranges. Positive associations are shown in navy and negative associations in terracotta.

The main synthetic paths follow the proposed trust-transfer and risk-dampening logic. The largest positive association is IT → AST ($\hat{\beta }=0.607$), placing AI service trust at the center of the transfer from institutional confidence to acceptance (Figure 3). The negative IT → RP path ($\hat{\beta }=-0.271$) and the stronger negative RP → AST path ($\hat{\beta }=-0.459$) locate risk perception within the same mechanism: institutional trust is associated with lower perceived risk, and perceived risk reduces confidence in the AI service. The positive AST → BI path ($\hat{\beta }=0.424$) identifies service-level trust as the closest condition for intended use. The smaller residual IT → BI path ($\hat{\beta }=0.284$) leaves institutional confidence with a background association after service trust is considered. The narrow seed ranges indicate stable path directions across the three simulation runs.

The descriptive indirect products clarify how the two routes differ in size (Table 2).

**Table 2:** Descriptive indirect products in the synthetic simulation.

Indirect path	Product	Direction
IT → RP → AST	+0.124	+
IT → AST → BI	+0.257	+
IT → RP → AST → BI	+0.053	+

The IT → AST → BI product is +0.257, larger than the longer IT → RP → AST → BI route (+0.053). The service-trust route is therefore the stronger path from institutional trust to intended use in the synthetic mechanism. The positive IT → RP → AST product (+0.124) arises because institutional trust is linked to lower risk perception, and lower risk perception is linked to higher service trust. The two products distinguish a direct trust-transfer route from a risk-reduction route.

**Figure 4:**
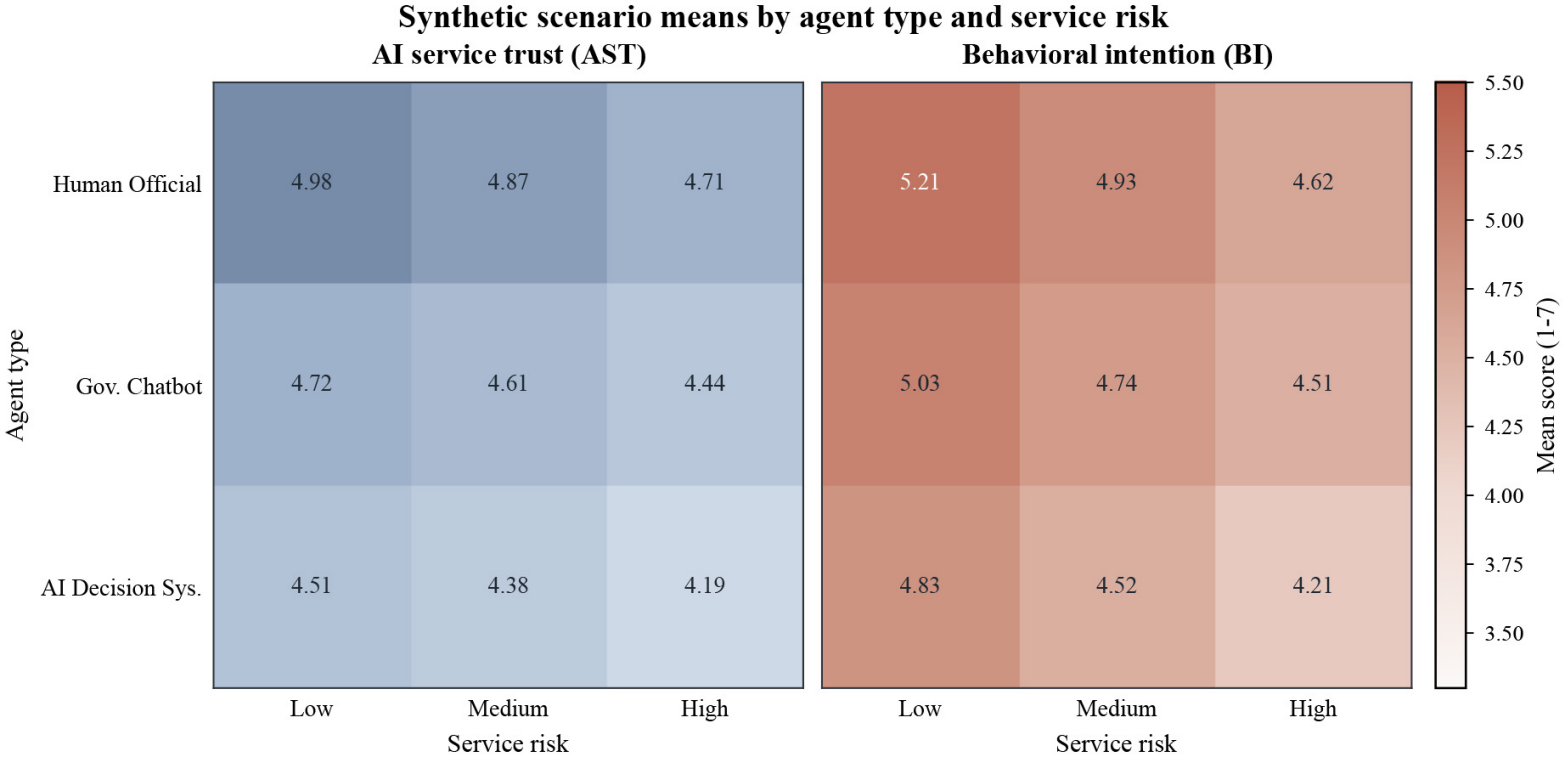
Synthetic scenario means by agent type and service risk. Heatmaps report mean AI service trust (AST) and behavioral intention (BI) on the 7-point response scale. Darker cells indicate higher means.

Service format changes the strength of trust transfer across the scenario cells (Table 3; Figure 4). For the IT → AST path, coefficients are lowest in the AI Decision System condition at low, medium, and high risk, showing weaker transfer from institutional confidence to service trust when decision support is fully automated. The Government Chatbot condition has the highest IT → AST coefficients across all three risk levels, consistent with stronger institutional anchoring through government branding, an interactive interface, and possible escalation.

**Table 3:** Cell-level coefficients from the synthetic 3 × 3 scenario experiment (*n* = 100 per cell).

Agent type	Risk level	IT → AST	AST → BI
Human official	Low	0.584	0.401
Human official	Medium	0.571	0.418
Human official	High	0.558	0.443
Gov. Chatbot	Low	0.612	0.398
Gov. Chatbot	Medium	0.598	0.467
Gov. Chatbot	High	0.579	0.526
AI decision sys.	Low	0.571	0.412
AI decision sys.	Medium	0.543	0.451
AI decision sys.	High	0.521	**0.332**

Bold indicates the weakest trust-to-adoption conversion in the high-risk condition.

The AST → BI coefficients identify where service trust converts less readily into intended use. Under high risk, the AI Decision System has the weakest AST → BI coefficient (0.332), whereas the Gov. Chatbot condition retains a higher conversion coefficient (0.526) and the Human Official condition sits between them (0.443). The H5 pattern appears at this translation step: service trust remains present, but high consequence and full automation weaken its movement into willingness to use. The government-chatbot pattern also indicates that institutional branding and possible human escalation preserve part of the trust-to-use pathway in consequential tasks.

The scenario means show the same contextual pattern at the level of trust and intention. Within each agent type, both AST and BI decline as service risk moves from low to high, indicating that higher service stakes raise the acceptance threshold across both outcomes. Within the same risk level, the AI Decision System generally sits below the Human Official and Gov. Chatbot formats. Across all nine cells, the lowest values concentrate in the fully automated high-risk condition (AST = 4.19; BI = 4.21). In that high-risk condition, the AST → BI coefficients for Human Official, Gov. Chatbot, and AI Decision System are 0.443, 0.526, and 0.332, respectively. The fully automated decision system therefore marks the clearest acceptance constraint, consistent with an *algorithmic aversion ceiling* in which high consequence and complete automation limit the conversion of service trust into intended use (Dietvorst et al., 2015; Logg et al., 2019; Horvath et al., 2023).

The temperature results retain the same path directions across *T* = 0.3, *T* = 0.7, and *T* = 1.0 (Table 4). At *T* = 0.3, coefficients are larger because lower temperature compresses response dispersion. At *T* = 1.0, magnitudes decline while signs remain unchanged. The trust-transfer and risk-dampening directions are therefore stable across the tested generation temperatures.

**Table 4:** Temperature sensitivity of main path coefficients (*n* = 200 per condition).

Temperature	IT → AST	IT → RP	RP → AST	AST → BI	Deviation from main (%)
*T* = 0.3 (low)	0.836	−0.389	−0.621	0.571	+38, +44, +35, +35
*T* = 0.7 (main)	0.607	−0.271	−0.459	0.424	Baseline
*T* = 1.0 (high)	0.541	−0.231	−0.398	0.381	−11, −15, −13, −10

Deviations are reported relative to the main-temperature estimates in the order IT → AST, IT → RP, RP → AST, and AST → BI. Directions remain unchanged across settings, but lower temperature mechanically inflates coefficient magnitude by reducing response variance.

### 4.3 Human-validation pilot

The human-validation pilot provides a second view of the same trust mechanism in observed responses. Among 189 submitted records, 182 passed the attention check. The valid sample has a mean age of 27.75 years (SD = 5.05), includes 107 female, 70 male, and 5 non-disclosed participants, and consists of public-administration-related graduate or MPA students. Residence origin is 56.0% urban, 24.7% county or town, and 19.2% rural. Mean prior e-government use is 4.984 on the 7-point scale, mean AI familiarity is 4.388, and mean self-rated digital literacy is 4.873. The profile captures digitally experienced public-service users for the validation comparison.

The pilot scale descriptives show a response profile with clear variation across the four constructs (Table 5). Institutional trust remains above the midpoint, risk perception is clearly present, and AI service trust and behavioral intention form their own mid-range judgments. Cronbach's α values range from 0.889 to 0.943, supporting internal consistency in this pilot sample and giving a measurement basis for the path comparison.

**Table 5:** Human-validation pilot scale descriptives and reliability (*n* = 182).

Construct	Items	Mean	SD	Cronbach's α
Institutional trust (IT)	5	4.895	0.691	0.889
Risk perception (RP)	4	3.676	0.907	0.913
AI service trust (AST)	6	4.595	0.886	0.943
Behavioral intention (BI)	3	4.586	1.002	0.911

The standardized pilot regressions preserve the expected path directions while shifting weight toward service-level judgments (Table 6).

**Table 6:** Standardized associations in the human-validation pilot (*n* = 182).

Path	Model specification	$\hat{\beta }$	Direction
IT → AST	AST regressed on IT and RP	+0.357	+
IT → RP	RP regressed on IT	−0.240	−
RP → AST	AST regressed on IT and RP	−0.513	−
AST → BI	BI regressed on AST and IT	+0.650	+
IT → BI	BI regressed on AST and IT	+0.137	+

AST → BI is stronger in the pilot (β = 0.650) than in the synthetic path estimate ($\hat{\beta }=0.424$), indicating that observed use intentions are closely tied to the service-level trust judgment. RP → AST is also stronger in the pilot (β = −0.513 versus $\hat{\beta }=-0.459$), giving perceived risk a prominent role in human evaluations of service trust. The institutional-trust paths are more moderate in the human sample (IT → AST β = 0.357 and IT → RP β = −0.240). IT remains positively associated with BI after AST is included (β = 0.137), while behavioral intention is mainly organized around AI service trust.

**Figure 5:**
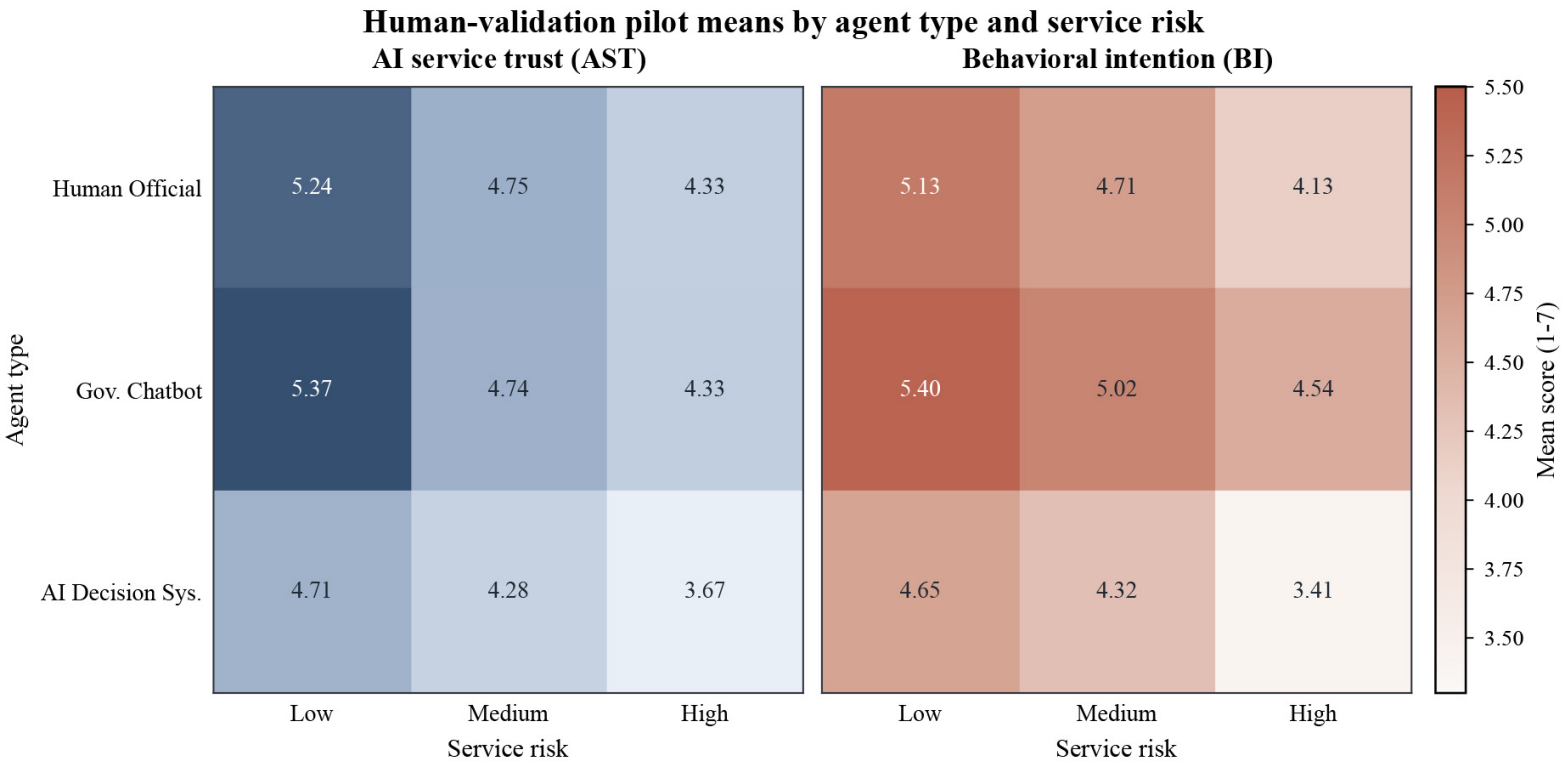
Human-validation pilot means by agent type and service risk. Heatmaps report mean AI service trust (AST) and behavioral intention (BI) on the 7-point response scale. Valid cell sizes range from 18 to 21. Darker cells indicate higher means.

At the scenario level, the human pilot preserves the synthetic ordering across the same nine cells. Synthetic and pilot means are strongly aligned: *r* = 0.803 for AST (*p* = 0.009) and *r* = 0.875 for BI (*p* = 0.002). Spearman rank correlations are 0.850 for AST and 0.867 for BI. The fully automated high-risk condition has the lowest pilot AST mean (3.667) and BI mean (3.413), whereas the government-chatbot low-risk condition has the highest pilot BI mean (5.400) (Figure 5).

The pilot means reproduce the main scenario ordering. The fully automated high-risk condition is lowest on both AST and BI, showing simultaneous weakening of service trust and intended use. The government-chatbot low-risk condition has the highest BI mean, indicating greater acceptance of a government-branded, interactive AI interface when task stakes are low. Human Official and Gov. Chatbot conditions are close in several low- and medium-risk cells, so the acceptance barrier is concentrated in the combination of substantial consequences and limited visible recourse. The high-risk AI Decision System cell therefore gives the clearest human-pilot counterpart to H5.

## 5 Discussion

The evidence suggests that institutional trust enters AI-enabled public service acceptance through a renewed judgment at the service interface. Earlier e-government research treated trust in public agencies as a basis for citizens' willingness to use online services (Parent et al., 2005), while trust-transfer theory explains how confidence in a familiar source can shape evaluations of a less familiar target (Stewart, 2003). The present pattern narrows that broader account for government AI. Institutional confidence is re-evaluated when citizens face a particular automated service and ask whether the system fits the administrative task, whether its operation can be explained, whether errors can be corrected, and whether responsibility remains visible. Recent work on public confidence in government AI places transparency, ethics, and perceived benefits at the center of trust formation (Bian et al., 2025); the contribution here is to locate those cues in the concrete service encounter where institutional trust is converted into AI service trust.

Perceived risk in this study is better understood as administrative consequence risk than as a general worry about technology. In information-systems research, perceived risk has long been associated with lower willingness to adopt electronic services (Featherman and Pavlou, 2003). Public-sector AI changes the content of that risk. The scenarios that depress AI service trust are those in which automated outputs may affect eligibility, enforcement support, complaint handling, correction opportunities, or the attribution of responsibility. This explains why risk weakens service-level trust even when institutional trust remains present. The result also sharpens recent work on algorithmic government by showing where fairness concerns and preferences for accountable AI are likely to enter the acceptance process: citizens evaluate system capability together with the contestability and governability of administrative consequences (Sidhu et al., 2024; Feng and Chandra, 2025).

Differences among human officials, government chatbots, and fully automated decision systems indicate that service format carries procedural meaning in public administration. Human involvement is read as a cue about reviewability, escalation, and the visibility of the administrative chain. A government chatbot can retain some of those cues when it is clearly identified with an agency, limited to a bounded task, and connected to possible human assistance. Recent work on public encounters and government chatbots similarly treats chatbot use as a change in citizen–state interaction, with the human element of public service delivery remaining analytically relevant (Alishani et al., 2026). The weaker conversion of AI service trust into behavioral intention under fully automated high-risk conditions is likely to reflect the loss of visible review and recourse. Citizens may regard an AI system as capable, yet hesitate to rely on it when the service outcome is consequential and the path back to administrative responsibility is unclear.

The practical implications follow from these distinctions across task type and accountability visibility. Routine information inquiry, appointment guidance, and procedural navigation can be served through automated or chatbot interfaces when the agency identity, task boundary, and handoff route are clear. Services involving eligibility screening, welfare access, enforcement support, complaints, correction, or appeal require a different design logic. In those settings, human review, responsibility statements, understandable explanations, and accessible appeal paths are part of the service infrastructure, not peripheral safeguards. Public-sector AI scholarship often emphasizes transparency, ethics, and institutional safeguards in general terms (Bian et al., 2025; Feng and Chandra, 2025); the present findings specify how those safeguards become usable at the point where citizens encounter automation.

The evidence structure combines controlled scenario comparison with a human-validation pilot to trace the proposed mechanism across two forms of response data. The synthetic simulation was used to formalize the trust-transfer, risk-dampening, and scenario-boundary assumptions under comparable service-format and risk-level conditions. The pilot responses offered a directional check on whether the same ordering appeared in human evaluations, strengthening the interpretation of the scenario pattern. Recent methodological discussions treat LLM-generated samples as useful tools for theory-oriented comparison and hypothesis refinement when their analytical role is clearly defined (Bisbee et al., 2024). In this study, the alignment between synthetic ordering and pilot responses provides a structured basis for subsequent empirical tests across more heterogeneous user groups, additional public-service domains, and institutional arrangements for procurement, data sharing, review, and appeal.

## 6 Conclusion

In AI-enabled public services, acceptance depends on how institutional confidence, perceived risk, and service-level trust are organized within the service encounter. High-risk automation is most vulnerable when review and recourse are difficult to see, since service trust then converts less readily into willingness to use. Institutional trust, risk perception, and AI service trust link public-sector AI acceptance to institutional responsibility and service design. Visible human review, responsibility allocation, and appeal channels help institutional confidence travel into trust in an AI-enabled service, especially when automated decisions carry consequences for citizens. For digital government, the practical task is to keep accountability visible where citizens encounter automation, particularly in services involving eligibility, complaints, enforcement, or other consequential administrative tasks.

## Data Availability

The raw data supporting the conclusions of this article will be made available by the authors, without undue reservation.
